# Review of epidemiological features, microbiological diagnosis and treatment outcome of microbial keratitis: Experience of over a decade

**DOI:** 10.4103/0301-4738.53051

**Published:** 2009

**Authors:** Usha Gopinathan, Savitri Sharma, Prashant Garg, Gullapalli N Rao

**Affiliations:** Jhaveri Microbiology Centre, Prof. Brien Holden Eye Research Centre, Hyderabad Eye Research Foundation, L.V. Prasad Eye Institute, Hyderabad, India; 1 Cornea Services, L. V. Prasad Eye Institute, Hyderabad, India

**Keywords:** Diagnosis, epidemiology, infective keratitis, outcome, treatment

## Abstract

**Purpose:**

To review the epidemiological characteristics, microbiological profile, and treatment outcome of patients with suspected microbial keratitis.

**Materials and Methods:**

Retrospective analysis of a non-comparative series from the database was done. All the patients presenting with corneal stromal infiltrate underwent standard microbiologic evaluation of their corneal scrapings, and smear and culture-guided antimicrobial therapy.

**Results:**

Out of 5897 suspected cases of microbial keratitis 3563 (60.4%) were culture-proven (bacterial – 1849, 51.9%; fungal – 1360, 38.2%; *Acanthamoeba* – 86, 2.4%; mixed – 268, 7.5%). Patients with agriculture-based activities were at 1.33 times (CI 1.16–1.51) greater risk of developing microbial keratitis and patients with ocular trauma were 5.33 times (CI 6.41–6.44) more likely to develop microbial keratitis. Potassium hydroxide with calcofluor white was most sensitive for detecting fungi (90.6%) and *Acanthamoeba* (84.0%) in corneal scrapings, however, Gram stain had a low sensitivity of 56.6% in detection of bacteria. Majority of the bacterial infections were caused by *Staphylococcus epidermidis* (42.3%) and *Fusarium* species (36.6%) was the leading cause of fungal infections. A significantly larger number of patients (691/1360, 50.8%) with fungal keratitis required surgical intervention compared to bacterial (799/1849, 43.2%) and *Acanthamoeba* (15/86, 17.4%) keratitis. Corneal healed scar was achieved in 75.5%, 64.8%, and 90.0% of patients with bacterial, fungal, and *Acanthamoeba* keratitis respectively.

**Conclusions:**

While diagnostic and treatment modalities are well in place the final outcome is suboptimal in fungal keratitis. With more effective treatment available for bacterial and *Acanthamoeba* keratitis, the treatment of fungal keratitis is truly a challenge.

There are several reports in literature, from different continents of the world, describing the prevalence of bacterial, fungal and parasitic pathogens in ulcerated corneas. [1–6] With the exception of a few population-based studies,[7,8] the majority of these reports, such as those from south Florida,[1] Nepal,[2] Bangladesh,[3] Ghana,[4] and India,[5,6] have primarily evaluated predisposing factors and causative agents of microbial keratitis in patients seen in the hospital. The number of patients in these studies has been less than 500 seen over a period of less than two years. Similarly, several publications on the management and treatment outcomes from various parts of the world are confined to certain groups of organisms in a limited number of patients with microbial keratitis.

In order to determine the impact of various epidemiological patterns, diagnostic methods and treatment strategies on the outcome of infective keratitis, results from studies employing standard procedures over a considerable period of time in a large number of patients would be most informative. At the L.V. Prasad Eye Institute, Hyderabad, India, every patient who reports to the cornea clinic with a stromal infiltrate in the cornea undergoes a standard protocol of clinical evaluation, diagnostic investigation, and therapeutic regimen, and all clinical and microbiological data is collected systematically.

The purpose of this study was to evaluate data pertaining to 5897 cases of presumed microbial keratitis investigated at this hospital over a period of 10 years and five months. We determined the factors predisposing to bacterial, fungal, *Acanthamoeba* and mixed infections, identified the causative agents prevalent, and analyzed the treatment outcome in patients with microbial keratitis.

## Materials and Methods

A search of the computerized corneal ulcer database showed that 5897 clinically suspected cases of infectious keratitis had undergone microbiological investigation at this referral eye care center between February 1991 and June 2001. All these cases were defined clinically as ‘corneal ulcers’, following observation of an epithelial defect overlying a stromal infiltrate as seen on slit-lamp biomicroscopic examination. Among the 5897 cases, the medical and the microbiology data of 3563 culture-proven cases of bacterial, fungal, *Acanthamoeba*, and mixed keratitis were reviewed to study the demographic features, possible predisposing factors, duration of symptoms, prior therapy received, seasonal variation and laboratory results. Treatment outcome was analyzed in all patients except those with mixed infections and patients lost to follow-up.

At presentation to the cornea services of this institute, information pertaining to demographic features, duration of symptoms, risk factors and occupational status was documented for every suspected case of infectious keratitis according to a detailed protocol. Cornea evaluation was carried out by a cornea specialist using a slit-lamp biomicroscope and findings were recorded in a predesigned format. Detailed diagrammatic documentation of the ulcer was done and recorded on a daily basis. Treatment regimen, response to treatment and final outcome were recorded in all cases.

Following clinical examination, patients were subjected to microbiological investigations as per the institutional protocol described earlier.[9,10]

The bacterial and fungal isolates were identified up to the species level using standard microbiological procedures.^11^ The smear and culture results were recorded in the predesigned format along with clinical details and captured in the corneal ulcer database which is maintained systematically. All analysis projected in this study is derived from this database.

The standard protocol used for the treatment of our patients is described in detail in an earlier publication which reported microbial keratitis in an elderly population seen at this institute from February 1991 until June 1995.[10] The treatment protocol has remained unchanged since then for bacterial and fungal keratitis, however, we have adopted combined therapy with polyhexamethylene biguanide (0.02%) and chlorhexidine (0.02%) for *Acanthamoeba* keratitis since August 1996.[12] Surgical mode of treatment included tissue adhesive application with bandage contact lens, penetrating keratoplasty, evisceration, whenever applicable. Treatment outcome at the end of three months or at the completion of treatment (whichever was earlier) was considered for analysis.

### Statistical analysis

Student's t test was applied to compare the mean values of demographic factors such as age. The chi square test was used for comparison of proportions. The odds ratio (OR) with 95% confidence interval (CI) was employed to assess the relative risk of patients with trauma and agriculture-related occupation developing microbial keratitis.

## Results

**Figure 1:**
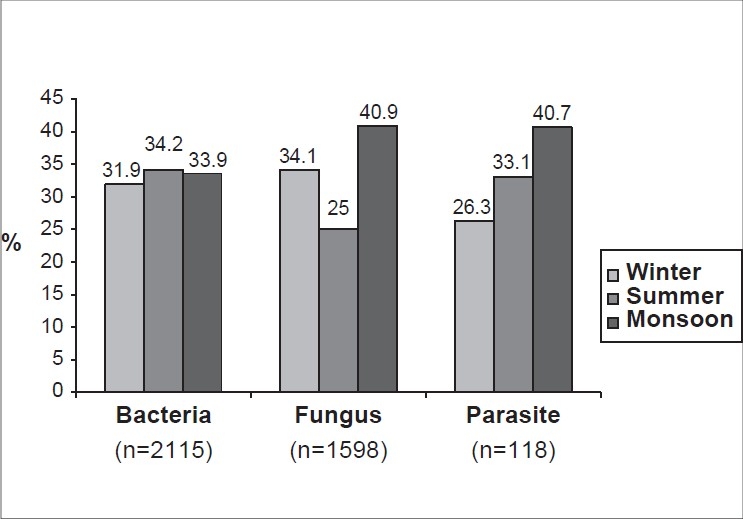
Seasonal variation in the occurrence of microbial keratitits (includes pure and mixed cases) in southern India

Of the 5897 clinically suspected cases of infectious keratitis, 4087 (69.3%) were males and 1810 (30.7%) were females, the overall male to female ratio of patients being 2.25:1. Laboratory evidence of microbial infection was established in 3563 (60.4%) of 5897 cases whose corneal scrapings were subjected for smears and culture. The mean (± standard deviation) age was 41.20 (± 20.36) years in patients with bacterial keratitis (1849, 51.9%), 30.90 (± 15.28) years in patients with fungal keratitis (1360, 38.2%), and 34.45 (± 12.54) years in patients with *Acanthamoeba* keratitis (86, 2.4%), indicating a relatively increased occurrence of corneal infections (irrespective of the etiological agent) in the middle age group. The seasonal variation in the occurrence of all (including mixed) bacterial, fungal and *Acanthamoeba* keratitis is as depicted in Fig. 1.

Unilateral ulcer cases included 1789 right eyes and 1737 left eyes. Thirty-seven patients had bilateral infection accounting for 3600 affected eyes. Since both eyes of patients with bilateral infection revealed identical organisms, the occupational status, possible risk factors, duration of symptoms, prior medication, and laboratory parameters were analyzed taking into account 3563 patients and not eyes.

The occupations of patients [Table 1] were classified as outdoor (agriculture and manual labor), and indoor (desk job and household). More number of patients with fungal, *Acanthamoeba* (pure cultures) and polymicrobial keratitis (bacteria and fungus; bacteria and parasite) were found to be involved in agriculture-related activities (*P* < 0.001) as compared to other occupations; this feature was not evident in patients with pure bacterial keratitis and in cases where fungus and *Acanthamoeba* coexisted. Odds ratio (OR) revealed that patients involved in agriculture-based activities were 1.33 times (CI 1.16-1.51) at a greater risk of developing microbial keratitis.

**Table 1:** Occupational status of patients with microbial keratitis (n = 3448)

Occupation	No. (%)	Fungal No. (%)	Parasitic No. (%)	B + F No. (%)	B + P No. (%)
Agriculture	344 (19.3)	366 (27.7)*	29 (34.5)*	75 (32.8)*	13 (43.3)*
Manual labor	337 (18.9)	269 (20.3)	18 (21.4)	56 (24.5)	4 (13.3)
Desk Job	191 (10.7)	174 (13.2)	4 (4.8)	28 (12.2)	2 (6.7)
Household	312 (17.5)	223 (16.9)	17 (20.2)	25 (10.9)	4 (13.3)
Unemployed	599 (33.6)	290 (22.0)	16 (19.0)	45 (19.7)	7 (23.3)
Total	1783	1322	84	229	30

B–Bacterial; F–Fungal; P–Parasitic; Data not available for 115 cases;

* *P* < 0.001

The potential predisposing ocular factors identified in patients are shown in Table 2. Between the three etiological groups (pure cultures), the association of trauma was more pronounced for fungal and parasitic keratitis as compared to bacterial (*P* < 0.001). Overall, patients with ocular trauma were 5.33 times (CI 6.41-6.44) at a greater risk of developing microbial keratitis.

**Table 2:** Predisposing ocular factors in microbial keratitis (n=2881)

Factors	Bacterial No. (%)	Fungal No. (%)	Parasitic No. (%)	Bacterial + Fungal No. (%)
*Trauma	838 (46.6)	712 (81.9)	42 (95.5)	122 (71.7)
Prior surgery	394 (22.0)	83 (9.5)	0 (0)	21 (12.4)
Corneal scar	113 (6.3)	19 (2.2)	0 (0)	3 (1.8)
Epithelial defect	111 (6.2)	14 (1.6)	1 (2.2)	8 (4.7)
Corneal edema	100 (5.6)	4 (0.5)	0 (0)	2 (1.2)
Dry eye	62 (3.4)	0 (0)	0 (0)	0 (0)
Glaucoma	53 (2.9)	8 (0.9)	0 (0)	1 (0.6)
Lagophthalmos	51 (2.8)	15 (1.7)	0 (0)	8 (4.7)
Blepharitis	40 (2.2)	11 (1.8)	0 (0)	3 (1.8)
Contact lens wear	36 (2.0)	3 (0.3)	1 (2.3)	2 (1.2)
Total	1798	869	44	170

Data not available for 682 cases;

* *P* < 0.001

**Figure 2:**
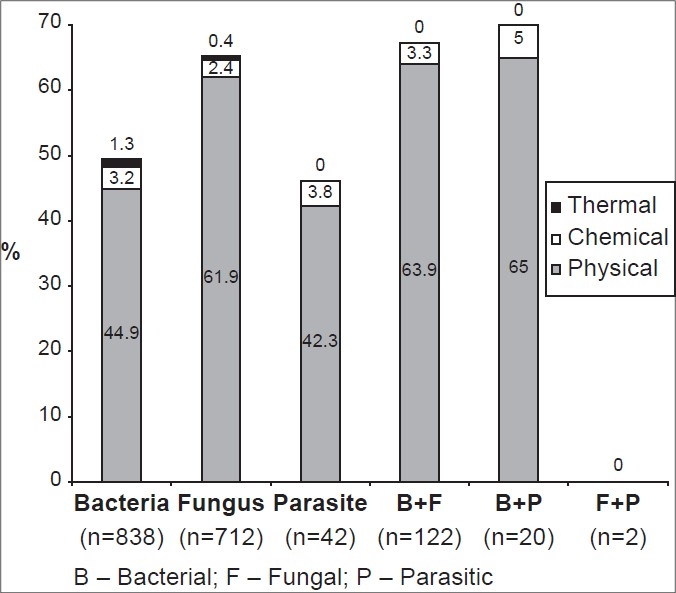
Agents causing trauma in patients with microbial keratitis

Patients with outdoor occupation had higher prevalence of keratitis due to trauma as compared to the patients engaged indoors. This observation was significant for bacterial (*P* < 0.001), fungal (*P* < 0.001) and *Acanthamoeba* (*P* = 0.02) keratitis when all culture-positive trauma and non-trauma cases were considered. In keratitis of pure or polymicrobial origin, physical agents were the most frequent sources of corneal injury than the other two (*P* < 0.001) as depicted in Fig. 2. Among the systemic factors documented in 296 patients, diabetes mellitus was more frequently noted in keratitis of both pure and polymicrobial etiology, accounting for 69.2% (205/296) cases.

Among the 3563 patients, 1945 (54.6%) were treated with antimicrobial agents and corticosteroids topically elsewhere, prior to their presentation to our cornea services [Table 3]. When retrospectively analyzed it was observed that in 945 (48.6%) patients the antimicrobial agents received were partly or completely in agreement with the type of the microbial agent (bacterial or fungal) causing the infection as proven by culture. Most patients, however, had received the medications in less than optimum dosage.

**Table 3:** Treatment received by the patients prior to presentation at the institute

Type of keratitis	No.	Appropriate antimicrobial therapy	Indiscriminate combination therapy*	Antibiotics with steroids
Bacterial	907	405 (AB)	331	171
Fungal	825	415 (AF)	395	15
Parasitic	50	0	50	0
B + F	147	60 (AB)	20	9
		14 (AF)		
		44 (AF+AB)		
B + P	15	7 (AB)	7	1
F + P	1	0	1	0
Total	1945	945 (48.4%)	804 (41.3%)	196 (10.0%)

AB – Antibiotic; AF – Antifungal; B – Bacteria; F – Fungus; P–Parasite,

* Antiviral, Antibiotic, Antifungal

**Figure 3:**
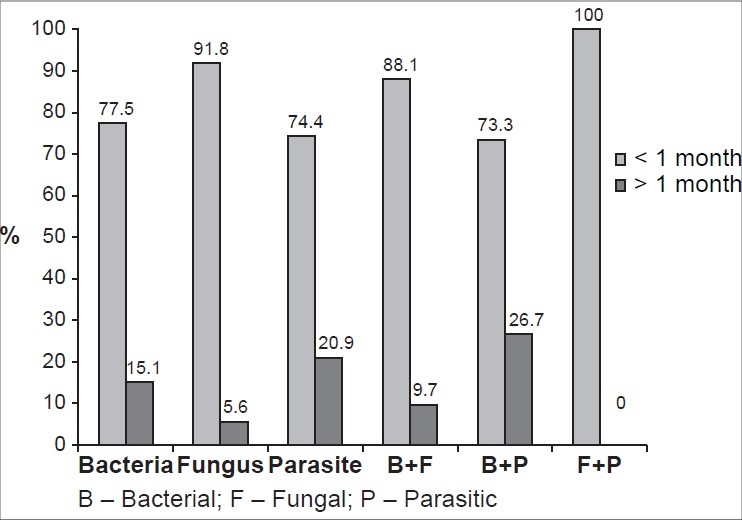
Duration of symptoms prior to presentation at the institute (n=3382)

Overall, greater number of patients had sought medical help at our institute with duration of symptoms less than one month (2977) than those with symptoms longer than one month (405) as shown in Fig. 3 (*P* < 0.001). One thousand two hundred and fifty-two (0.06%) of 2977 patients had visited the institute within one week of onset of symptoms. On the whole, lower socioeconomic group patients (non-paying) consisted of a greater segment of the patients with microbial keratitis (3255/5897, 55.1%) as well as with positive cultures (2050/3563, 57.5%).

Direct microscopic examination of corneal scrapings detected microbes in 2884 (80.9%) of 3563 culture-positive cases. Overall, culture was positive for bacteria in 2115 (59.3%), for fungi in 1598 (44.8%) and *Acanthamoeba* in 118 (3.3%) of all cases (pure and polymicrobial cases). The smears revealed bacteria in 62.5% (1325/2115), fungi in 94.6% (1511/1598) and *Acanthamoeba* in 85.6% (101/118) of the cases. The sensitivity and specificity of each of the staining techniques employed in the detection of bacteria, fungi and *Acanthamoeba* are given in Table 4. On analysis of matching smear and culture results, Gram stain was accurate in only 45.7% of the corneal scrapings from 2115 patients with bacterial keratitis (pure and mixed). Among the 2334 culture-negative cases, smears were positive for microorganisms in 739 (31.7%) cases revealing bacteria in 417 (17.9%), fungus in 298 (12.8%), *Acanthamoeba* in 19 (0.8%) and both bacteria and fungus in five (0.2%) eyes. These cases being culture-negative were not analyzed in this study.

**Table 4:** Sensitivity and specificity of corneal scraping smears in the detection of microorganisms with culture as gold standard

Smears n*	Bacteria		Fungi		*Acanthamoeba*	
			
	Sensitivity %	Specificity %	Sensitivity %	Specificity %	Sensitivity %	Specificity %
Gram 3442	56.6	97.8	89.8	93.7	73.3	99.8
Giemsa 2774	ND	ND	85.2	96.1	72.2	99.8
KOH + CFW 2555	ND	ND	90.6	94.3	84.0	99.8

A – Sensitivity; B – Specificity; ND – Not Done; KOH+CFW – potassium hydroxide + calcofluor white,

n* is different for each staining method as all procedures could not be done for all cases

Of 3563 cases of microbial keratitis, 3295 (92.5%) revealed pure growth of either bacteria (1849, 51.9%), fungi (1360, 38.2%) or *Acanthamoeba* (86, 2.4%). Polymicrobial infection was seen in 268 (7.5%) cases. Of the 37 cases that presented with bilateral infection, 34 cases demonstrated pure bacterial growth, two had pure fungal growth, and one had mixed infection of both bacteria and fungus, both eyes of each of these patients revealing similar organisms.

More than one bacterium (two or more) was isolated from 350 cases resulting in 2511 bacterial isolates. Among the bacterial isolates, 2062 (82.1%) were gram-positive and 449 (17.9%) were gram-negative. The different bacterial and fungal species isolated are listed in Table 5 and [6] respectively. *Propionebacterium* (19, 0.8%) and *Peptostreptococcus* (seven, 0.3%) species were the only anaerobes recovered in this series. The antibiotic susceptibility data of the bacterial isolates is beyond the scope of this study and is published elsewhere.[13,14] Overall, 1648 fungal isolates were recovered from culture of corneal scrapings (50 patients had more than one isolate). Of these, 1635 (99.2%) were molds and 13 (0.8%) were yeasts.

**Table 5:** Types of bacterial species isolated in patients with microbial keratitis (n = 2511)

Organism	No. (%)
Gram-positive cocci	1594 (63.5)
*Staphylococcus epidermidis*	817 (32.5)
*Staphylococcus aureus*	134 (5.3)
*Staphylococcus* spp.	112 (4.5)
*Streptococcus pneumonias*	348 (13.9)
*Streptococcus* spp.	163 (6.5)
Other cocci	13 (0.5)
Peptostreptococcus	7 (0.3)
Gram-positive bacilli	468 (18.6)
*Corynebacterium* spp.	363 (14.5)
*Nocardia* spp.	46 (1.8)
*Bacillus* spp.	28 (1.1)
*Propionebacterium* spp.	19 (0.8)
*Mycobacterium* spp.	10 (0.4)
Other bacilli	2 (0.1)
Gram-negative cocci	9 (0.4)
*Branhamella catarrhalis*	4 (0.2)
*Neisseria* spp.	4 (0.2)
*Brevibacterium* spp.	1 (0.03)
Gram-negative bacilli	440 (17.5)
*Pseudomonas aeruginosa*	244 (9.7)
*Pseudomonas* spp.	58 (2.3)
*Moraxella* spp.	36 (1.4)
*Haemophilus* spp.	24 (1.0)
*Acinetobacter* spp.	14 (0.6)
*Enterobacter* spp.	12 (0.5)
*Aeromonas* spp.	11 (0.4)
*Klebsiella* spp.	11 (0.4)
*Escherichia coli*	7 (0.3)
*Proteus* spp.	6 (0.2)
*Alkaligenes fecalis*	3 (0.1)
*Flavobacterium* spp.	3 (0.1)
Other gram-negative bacilli	11 (0.4)

Note: Multiple bacteria isolated from 350 cases

**Table 6:** Type of fungal species isolated in patients with microbial keratitis (n = 1648)

Organism	No. (%)
Hyaline fungi	1317 (79.9)
*Fusarium solani*	294 (17.8)
*Fusarium* spp.	294 (17.8)
*Aspergillus flavus*	299 (18.1)
*Aspergillus fumigatus*	103 (6.3)
*Aspergillus terreus*	30 (1.8)
*Aspergillus niger*	11 (0.6)
*Aspergillus* spp.	35 (2.1)
*Acremonium* spp.	18 (1.1)
*Chrysosporium* spp.	5 (0.3)
*Scedosporium apiospermum*	5 (0.3)
*Humicola* spp.	2 (0.1)
*Penicillium* spp.	2 (0.1)
*Phoma* spp.	1 (0.06)
*Rhizopus* spp.	1 (0.06)
Unidentified fungi	217 (13.2)
Molds Dematiaceous fungi	318 (19.3)
*Curvularia lunata*	47 (2.8)
*Curvularia* spp.	43 (2.6)
*Bipolaris* spp.	16 (1.0)
*Exserohilum* spp.	12 (0.7)
*Cladosporium* spp.	8 (0.5)
*Lasiodiplodia theobromae*	8 (0.5)
*Alternaria* spp.	5 (0.3)
*Torula* spp.	4 (0.2)
*Aureobasidium* spp.	2 (0.1)
*Nigrospora* spp.	1 (0.06)
*Epicoccum* spp.	1 (0.06)
Unidentified fungi	171 (10.4)
Yeasts	13 (0.8)
*Candida albicans*	1 (0.06)
Other *Candida* spp.	12 (0.7)

Note: More than one fungal isolate in 50 cases

All patients were started on medical therapy initially, however, 46.6% of the patients required surgical intervention as shown in Table 7. Overall treatment outcome in bacterial, fungal, and *Acanthamoeba* keratitis patients is shown in Table 8. Significantly more number of patients required surgical treatment in fungal keratitis compared to bacterial and *Acanthamoeba* keratitis.

**Table 7:** Surgical treatment received by 1662 culture-positive cases of microbial keratitis

		Types of Surgery		
Groups	n	TABCL (%)	PK (%)	Evisceration (%)
Bacterial	1849	432 (23.4)*	292 (15.8)	75 (4.1)
Fungal	1360	257 (18.9)	321 (23.6)*	113 (8.3)*
Parasitic	86	9 (10.5)	3 (3.5)	3 (3.5)
Bacterial + Fungal	236	52 (22.0)	68 (28.8)	27 (11.4)
Fungal + Parasitic	2	0 (0.0)	0 (0.0)	1 (50.0)
Bacterial + Parasitic	30	3 (10.0)	4 (13.3)	2 (6.7)
Total	3563	753	688	221

TABCL – Tissue adhesive with bandage contact lens, PK – Penetrating keratoplasty,

* *P* < 0.05

**Table 8:** Treatment outcome in patients with microbial keratitis (*n*=2729)

Outcome	Bacterial (n=1524) No. (%)	Fungal (n=1135) No. (%)	Acanthamoeba (n=70) No. (%)
Healed Scar	1151 (75.5)*	736 (64.8)	63 (90.0)*
Adherent leucoma	17 (1.1)*	3 (0.3)	1 (1.4)
No response / worsening	19 (1.3)	20 (1.8)	1 (1.4)
Evisceration	75 (4.9)	113 (10.0)*	3 (4.3)
Clear graft	59 (3.9)	90 (7.9)	1 (1.4)
Failed graft	105 (6.9)	102 (9.0)*	1 (1.4)
Graft Infiltrate	98 (6.4)	71 (6.3)	0 (0.0)

* *P* < 0.05, Note: Patients with mixed infection were not analyzed. Data was not available for 834 patients

## Discussion

A variety of factors determine clinical outcome in microbial keratitis and the epidemiological patterns vary from one country to the other and in different geographical areas in the same country. A comprehensive data is important to develop appropriate diagnostic and therapeutic strategies. This study reports the experience with 3563 culture-positive non-viral microbial keratitis patients based in southern India. The data reported here is expected to be useful in all areas of the world where fungal keratitis is relatively more prevalent and is commonly considered in the differential diagnosis of microbial keratitis.

The male preponderance in this series was observed not only in the overall clinically suspected cases of microbial keratitis but also in culture-proven cases of microbial keratitis (male:female:2.25:1, 2.24:1 respectively). Though both sexes develop corneal ulcers more commonly in the middle decades of life, a significant male preponderance has been reported by most previous studies[5,15] including those in children[2,16] and elderly patients.[2,11] Considering the predominant predisposing factor of trauma in all types of microbial keratitis (bacterial – 46.6%, fungal – 81.9%, *Acanthamoeba* – 95.5%) the probable reason for male preponderance is obvious. Ocular trauma was significantly more associated with outdoor occupation in this series.

More than half of the patients with culture-proven microbial keratitis (54.6%) had visited a physician prior to presentation at this institute and nearly half (48.6%, Table 3) of them had received antimicrobial agents that were appropriate, albeit on lower dosage, for the microbial agent involved. Therefore, we believe that despite the patient being on prior antimicrobial therapy, microbiological investigation may succeed in establishing etiological diagnosis in at least 50% of the patients. Traditional medicine or home remedy was used by only 0.4% of our patients compared to 37.3% of the patients in the study from Madurai.[5] The urban location of our institute in contrast to the semi-urban location of the institute at Madurai may account for this difference. While use of plant extracts has been reported from rural Malawi, Africa by Courtright *et al*.[17], it is fortunately not common in areas undergoing urbanization.

It is interesting to note that a majority of our patients presented within one month of onset of symptoms, 42% of whom came within one week. This indicates easy availability of transport to patients and is in contrast to the situation in other developing countries such as Nepal[2] where 19.3% of the patients took longer than one month to reach the hospital for treatment. Transport facilities and access to healthcare systems are important issues in the developing countries and our analysis points at optimum availability in the area catered by this institute.

Direct microscopic examination of corneal scrapings provides rapid diagnosis and forms the basis for instituting initial antimicrobial therapy which may be modified later according to culture reports.[18] An accurate smear diagnosis therefore becomes important in achieving optimum treatment outcome. The detection of fungi and *Acanthamoeba* was much higher in the smears than it was for bacteria [Table 4]. The detection rate for bacteria (Gram stain) was reduced by 10.9% when a correlation of the presence of similar bacteria in smears and cultures was made. We recently analyzed the utility of Gram stain in the diagnosis of early and advanced bacterial keratitis wherein the sensitivity was found to be 36.0% and 40.0% respectively.[19] The low sensitivity was attributed by us to the use of antibiotics prior to presentation at this institute by nearly 50% of the patients. The sensitivity of Gram stain in the diagnosis of bacterial keratitis, as reported by other authors (Asbell *et al.*[20] – 67%, Dunlop *et al.*[21] – 62%), is close to the overall sensitivity noted in this study (56.6%) which dropped on correlation of presence of similar bacteria in smears and cultures (45.7%).

Microorganisms were isolated in 60.4% of the 5897 cases of presumed microbial keratitis. This figure is close to many other reports[4,5] but is lower than the reports from Nepal[2] (80%) and from Bangladesh (81.7%).[21] The protocol of culture techniques followed in this study and the procedure of sample inoculation directly in the clinic leaves virtually no scope for role of laboratory-related reasons for low yield in culture. Patient-related causes such as prior antimicrobial therapy probably have a significant role to play, as has been suggested by Srinivasan *et al*.[5]

A majority of our patients (3295/3563, 92.5%) had corneal infection by a single agent, the most common being bacterial (1849/3563, 51.9%). Bacterial keratitides were predominantly caused by gram-positive bacteria. However, unlike other studies from Asia[2,5] and Africa[22] where infections by *Streptococcus pneumoniae* were most common; in our study, *Staphylococcus epidermidis*-related bacterial keratitis predominated. A review of literature showed that most of the studies from developed countries such as the USA[1,15,20] (except southern USA) and Australia[23] listed *S. epidermidis* or coagulase-negative staphylococci as the leading cause of bacterial keratitis. It is not clear whether the tendency to consider *S. epidermidis* or coagulase-negative staphylococci as a normal commensal of the conjunctiva may have led to underreporting in some of the studies. Nevertheless, the criteria to determine the significance of a positive culture from corneal scrapings appeared similar across most of these studies. Considering the fact that *S. epidermidis* forms the commonest commensal of the extraocular surfaces, it is highly probable that these organisms invade corneal tissues when compromised by antimicrobial and/ or corticosteroid therapy or trauma. The higher incidence of *S. pneumoniae* keratitis in Madurai compared to this series remains inexplicable since both these studies are from southern India. The strong association of chronic dacryocystitis with *S. penumoniea*-related microbial keratitis is well known[24] but the database in this study was not adequate to determine the frequency of concomitant sac pathology in our patients. It is possible that a larger number of patients with dacryocystitis were present in studies with predominant *S. pneumoniae* infection.

A high prevalence of fungal keratitis caused by filamentous fungi in warmer climates has been widely reported.[1,4,25] All cases (pure and polymicrobial) were considered together in this series; fungi were isolated in 1598 (44.8%) patients, a frequency similar to that reported from Madurai.[5] Some of the fungal isolates could not be definitely identified due to lack of characteristic spores [Table 6] in the medium used at our center for culturing fungus (Sabouraud dextrose agar, potato dextrose agar). Difficulty in speciation of fungi owing to lack of sporulation has been faced by other investigators as well.[1] Attempts were not made in this study to use spore-enhancing media for fungal isolates on a routine basis, which probably would have helped in speciation of some of the unidentified isolates.

The overall incidence of *Acanthamoeba* keratitis (3.3%) was low in this study although the number of affected patients was large (118). Only one patient had worn contact lenses (0.8%). In contrast to the literature from developed countries, where contact lens wear emerges as a great risk factor for developing infectious keratitis,[26,27] it accounted for only 42 out of 3563 (1.2%) cases in this series of which the majority (36/42, 85.7%) were bacterial. No patient among a series of 33 cases of *Acanthamoeba* keratitis, recently reported from south India, had worn contact lenses.[28] Concomitant infection with bacteria (0.8%) and fungi (0.1%) was quite rare in patients with *Acanthamoeba* keratitis. Diagnosis based on initial smear examination of corneal scraping was most rewarding in calcofluor white stained smears by fluorescence microscopy.

A significantly larger number of patients (691/1360, 50.8%) with fungal keratitis required surgical intervention compared to bacterial (799/1849, 43.2%) and *Acanthamoeba* (15/86, 17.4%) keratitis thus indicating a poor response to treatment in fungal keratitis compared to bacterial and *Acanthamoeba* keratitis (*P* < 0.05). This study shows that although bacterial and *Acanthamoeba* keratitis can be treated effectively, the treatment of fungal keratitis remains a challenge.
